# Mapping the global evidence base of bundibugyo ebolavirus disease: a systematic scoping review of research gaps and preparedness priorities

**DOI:** 10.1186/s12879-026-13977-1

**Published:** 2026-07-09

**Authors:** Abigail Boatemaa, Baffour Osei, Gabriel Osei Forkuo

**Affiliations:** 1 Faculty of Health Studies, University of Bradford, Richmond Road, Bradford, BD7 1DP UK; 2 University of Skills Training and Entrepreneurial Development (USTED), Kumasi, Ghana; 3 Department of Forest Engineering, Forest Management Planning and Terrestrial Measurements, Transilvania University of Brasov, Brasov, Romania

**Keywords:** Case fatality rate, Democratic republic of the congo, International concern, Monoclonal antibodies, Public health emergency of zoonotic reservoir, Risk of bias

## Abstract

**Background:**

Bundibugyo ebolavirus (BDBV) is a rare filovirus species with an estimated case fatality rate (CFR) ranging from 25% to 51%. The 2026 BDBV outbreak in the Democratic Republic of the Congo (DRC) was declared a Public Health Emergency of International Concern (PHEIC), highlighting the critical necessity of assessing global research preparedness for this pathogen.

**Objectives:**

To systematically map the global scientific evidence base on BDBV, characterize temporal and thematic publication trends, identify critical knowledge gaps, and formulate actionable priorities for public health response and clinical research.

**Methods:**

Following PRISMA-ScR guidelines, we programmatically searched PubMed/MEDLINE, OpenAlex, and Semantic Scholar for literature published from 2007 through May 2026. From 4,379 screened records, 100 high-relevance studies were selected using a weighted composite relevance scoring system. To structure the evidence, we synthesized a novel conceptual model mapping BDBV research across three core pillars—epidemiological drivers, health system responses, and public health outcomes—connected by a policy feedback loop and conditioned by cross-cutting systemic moderators (conflict, weak infrastructure, global governance, and research gaps). Methodological quality was evaluated using the Risk of Bias 2.0 (RoB 2) framework.

**Results:**

Only 23% of the included studies specifically addressed BDBV, with the remainder focusing on broader ebolavirus topics dominated by Ebola virus (EBOV). Applying the conceptual model revealed pronounced asymmetries across the research domains: the literature remains heavily concentrated within upstream epidemiological drivers and clinical features. Conversely, critical health system response domains lack validated tools; no licensed vaccine, specific therapeutic agent, or point-of-care rapid diagnostic test validated for BDBV currently exists. While cross-reactive monoclonal antibodies show preclinical efficacy, clinical-grade validation remains absent, and longitudinal survivor outcomes are sparsely documented. Systemic moderators, particularly weak health infrastructure and conflict, continue to compound these gaps. Methodological risk of bias was moderate, driven by missing outcome data (42% of studies) and selective reporting (41%).

**Conclusions:**

BDBV remains understudied relative to its epidemic potential. Sustained, internationally coordinated investments modeled on prior EBOV frameworks are required to transition from reactive to proactive research models.

**Clinical trial number:**

Not applicable

## Introduction

### Background

Bundibugyo ebolavirus (BDBV) is a phylogenetically distinct species within the genus *Orthoebolavirus*, family *Filoviridae*. First identified in November 2007 during a haemorrhagic fever outbreak in the Bundibugyo District of western Uganda, BDBV represents a genetically novel lineage that diverges substantially from the more extensively characterised *Ebola virus* (EBOV) and *Sudan virus* (SUDV) [1]. Its glycoprotein (GP), the primary target for neutralising antibodies and vaccine design, shares only approximately 55–60% amino acid identity with EBOV GP, precluding cross-protection from currently licensed vaccines such as Ervebo^®^ (rVSV-ZEBOV) and Zabdeno^®^/Mvabea^®^ [2, 3].

Prior to 2026, BDBV had caused two documented outbreaks. The first occurred in Bundibugyo District, Uganda, from August 2007 to February 2008, resulting in 131 confirmed, probable, and suspect cases, 37 deaths, and a CFR of approximately 25% [4, 5]. The second outbreak occurred in Isiro, Province Orientale, DRC in 2012, resulting in 38 laboratory-confirmed cases, 13 deaths, and a CFR approaching 51% [5–7]. Across these outbreaks, BDBV has consistently demonstrated a lower CFR than EBOV — which reaches up to 90% — but has remained associated with significant mortality and prolonged survivor morbidity [8, 9].

The epidemiological context shifted in 2026: on 5 May 2026, WHO issued a disease alert, and on 17 May 2026 formally declared a Public Health Emergency of International Concern (PHEIC) [10, 11]. As of June 2026, the outbreak had resulted in more than 1,000 confirmed cases and over 139 confirmed deaths across the DRC and neighbouring Uganda, prompting emergency mobilisation of research and international humanitarian response [12]. The 2026 PHEIC has underscored the profound inadequacy of the global BDBV knowledge base and exposed critical gaps in diagnostics, vaccine development, and therapeutics [13, 14].

Despite its epidemic potential, BDBV remains critically understudied relative to EBOV. No licensed vaccine, specific therapeutic, or point-of-care diagnostic exists for BDBV [15]. The two licensed vaccine regimens for EVD — both virus-vectored — were developed against EBOV and have not been validated for BDBV [2, 3]. Monoclonal antibody (mAb) therapies that demonstrated efficacy against EBOV similarly lack clinical validation for BDBV, though cross-reactive candidates have been identified [16–18]. Biosafety Level 4 (BSL-4) containment requirements have severely limited the volume of experimental work possible [19], and the rarity of outbreaks has constrained clinical data collection [9]. Furthermore, survivor cohort data specific to BDBV are extremely sparse: long-term follow-up studies documenting post-Ebola syndrome, immune sequelae, and psychosocial burden in BDBV survivors are only beginning to emerge [20, 21].

Structural immunology has contributed critical insights into broadly neutralizing antibody epitopes. Murin et al. [22] described the structural basis of pan-ebolavirus neutralization by an antibody targeting the glycoprotein fusion loop, characterizing a conserved vulnerability shared across multiple ebolavirus species. West et al. [23] subsequently identified the structural basis of pan-ebolavirus neutralization by a human antibody against a conserved, yet cryptic epitope, demonstrating that cryptic sites can serve as immunogenic targets for rational vaccine design. These structural findings have been complemented by computational approaches: [24] employed computational design of broad-spectrum Ebola antibodies through framework and complementarity-determining region synergistic optimization, demonstrating proof-of-concept for machine-learning-assisted antibody engineering. Additionally [25], reported engineered bispecific antibodies that achieved broad and potent protection against multiple ebolavirus species, suggesting that engineered antibodies may overcome the limitations of naturally derived monoclonal antibodies].

The social dimensions of BDBV outbreaks have equally received insufficient attention. Studies from the Bundibugyo District have documented the fear, stigma, and community disruption generated by the 2007–2008 outbreak, with lasting gendered and socioeconomic effects on affected households [21, 26]. These dimensions are operational determinants of response success: stigma impedes case detection, mistrust drives treatment refusal, and community resistance disrupts contact tracing. The 2026 outbreak is centred in Ituri — the same geographic footprint as the 2018–2020 EBOV epidemic — meaning affected communities carry institutional memory of prior responses. Responders must operationalize lessons from community engagement during previous DRC EBOV responses. Filovirus outbreak surveillance frameworks developed in the context of the Bundibugyo response identified persistent structural weaknesses — including delayed case detection, limited laboratory capacity, and inadequate community trust — that remain relevant to preparedness planning today [4, 27]. MacNeil et al. [28] provided a dedicated post-outbreak analysis of filovirus outbreak detection and surveillance drawing on lessons from the original Bundibugyo response, identifying specific gaps in laboratory confirmation capacity and case-reporting timeliness that persist as structural weaknesses in endemic settings.

Systematic scoping reviews provide a structured mechanism for mapping an evidence landscape, identifying coverage gaps, and generating actionable research priorities — functions that are especially critical in the context of an ongoing PHEIC. To our knowledge, no prior systematic scoping review has comprehensively addressed the BDBV evidence base across all research domains. This review was therefore designed to fill that gap.

### Aim, objectives, and research questions

#### Aim

This systematic scoping review aimed to map and synthesise the global evidence base on Bundibugyo virus disease (BVD), characterise temporal and thematic research trends across all ebolavirus literature relevant to BDBV, identify critical gaps, and generate actionable priorities for research and public health response.

#### Objectives

The specific objectives of this review were:

i. To map the global volume and thematic distribution of BDBV-related research from 2007 to 2026 across 12 pre-specified research domains.
ii. To characterise temporal trends in publication activity and identify periods of reactive versus sustained research investment in BDBV.
iii. To synthesise key findings in BDBV-specific epidemiology, virology, diagnostics, therapeutics, and vaccinology from the included studies.
iv. To assess the risk of bias across included studies using the RoB 2.0 framework.
v. To delineate critical evidence gaps and priority research directions for the international community in the context of the 2026 DRC PHEIC.

#### Research questions

This systematic scoping review was guided by the following research questions, framed using the PCC (Population–Concept–Context) framework:

- **RQ1**: What is the global volume and thematic distribution of published research on BDBV and related ebolavirus topics from 2007 to 2026, and how has this changed over time?
- **RQ2**: What are the documented epidemiological patterns, clinical features, and mortality outcomes of BDBV outbreaks?
- **RQ3**: What vaccine platforms, monoclonal antibody candidates, and antiviral therapeutics have been developed or evaluated with demonstrated or potential activity against BDBV?
- **RQ4**: What diagnostic tools and surveillance strategies have been applied or proposed for BDBV detection and outbreak management?
- **RQ5**: What are the key evidence gaps across all research domains, and what priority investments are needed to achieve preparedness for future BDBV outbreaks?

## Literature review

### Theoretical framework and related studies

#### The ebolavirus evidence landscape: a field defined by EBOV

The broader ebolavirus research field has been profoundly shaped by the epidemiological dominance of *Ebola virus* (EBOV), which has caused the largest and most frequent outbreaks — most notably the 2013–2016 West Africa epidemic, in which 28,610 people were affected and 11,308 died [29]. This concentration of outbreaks has driven a correspondingly disproportionate allocation of research funding, infrastructure, and scientific attention toward EBOV, at the expense of rarer but still clinically significant species such as BDBV. As Munyeku-Bazitama et al. [29] demonstrate, non-EBOV filoviruses — including BDBV — represent undercharacterised threats to global health security precisely because their infrequent outbreak history has been misread as a proxy for lower pandemic potential.

The historical record of ebolavirus outbreaks has been comprehensively described in the literature included in this review. Muyembe-Tamfum et al. [6] document the geographic and temporal distribution of EVD outbreaks in Africa from 1976 onwards, noting that outbreaks occur predominantly in the Congo and Nile basins and that BDBV — discovered in 2007 — added a new dimension to the known diversity of pathogenic ebolaviruses [6]. A companion veterinary-epidemiological perspective on Ebola virus outbreaks in Africa, tracing the historical record from 1976 to 2012, further contextualises the emergence of BDBV within the broader filovirus outbreak landscape of sub-Saharan Africa [30]. The meta-analytic work of Izudi and Bajunirwe [8], drawing on 42 outbreaks across 16 countries from 1976 to 2022, confirms that BDBV carries a substantially lower pooled CFR than EBOV (66.6%) but remains a significant cause of mortality, with CFR estimates across outbreaks ranging from 25% to 51% [8].

#### BDBV-specific research: sparse but foundational

The foundational BDBV-specific literature is built on a small number of high-impact studies. The virological discovery and phylogenetic distinctiveness of BDBV were first established by Towner et al. [1], with Wamala et al. [31] documenting the 2007 Uganda outbreak’s 192 suspect cases and a critical three-month diagnostic delay that allowed sustained transmission [31]. Roddy et al. [5] clinically characterized 26 hospitalized patients during this outbreak, noting key symptoms like non-bloody diarrhea, severe headache, and asthenia, alongside a 25% case fatality rate (CFR) [5], while Gupta et al. [4] identified that fatal BDBV infection was linked to high antigen burden and immune dysregulation rather than a cytokine storm [4]. The 2012 DRC outbreak extended this evidence base geographically; Kratz et al. [32] described clinical presentations in Isiro, DRC, noting a higher CFR of approximately 51% compared to the Ugandan event [5]. For this outbreak, Hulseberg et al. [6] reconstructed evolutionary dynamics using molecular epidemiology [6], and Albariño et al. [7] confirmed the first BDBV emergence in the DRC via whole-genome sequencing within a broader cluster of concurrent filovirus outbreaks [7]. Additionally, Choi et al. [33] mathematically modeled transmission dynamics to estimate reproductive numbers and serial intervals [33], while studies of pan-filovirus neutralizing antibody responses in Congolese survivors have revealed cross-reactive immunity with implications for BDBV protective correlates [34, 35].

#### Countermeasure research: vaccines, therapeutics, and diagnostics

A significant proportion of the BDBV-relevant literature addresses countermeasure development, though almost entirely at the pre-clinical stage. The rVSV-based vaccine platform — the basis for the licensed Ervebo^®^ vaccine — has been evaluated for BDBV cross-protection in multiple non-human primate (NHP) studies. Woolsey et al. [3] reviewed the landscape of panfilovirus vaccine platforms, while Malherbe et al. [36] demonstrated the efficacy of a single-dose intranasal panebolavirus vaccine in preclinical models. Falzarano et al. [37] demonstrated that a single immunisation with a monovalent rVSV-EBOV vaccine provided heterologous cross-protection against BDBV challenge in NHPs, achieving a 75% survival rate, demonstrating that heterologous protection is achievable against emerging ebolavirus strains [37]. Mire et al. [38] subsequently showed that rVSV-based vaccines expressing BDBV-specific glycoprotein did protect NHPs from lethal BDBV challenge, establishing proof-of-concept for BDBV-targeted rVSV vaccines [38]. Woolsey et al. [3] confirmed that a recombinant rVSV-based vaccine expressing BDBV GP provided post-exposure protection in NHPs, constituting the strongest pre-clinical evidence to date for a deployable BDBV vaccine. A dedicated study by Woolsey et al. [39] confirmed that rVSV-BDBV provides post-exposure protection specifically against Bundibugyo ebolavirus infection in NHPs, directly supporting its potential use as a post-exposure prophylactic in outbreak contexts.

In the therapeutic domain, King et al. [16] identified the cross-reactive monoclonal antibody BDBV223 — isolated from a human BDBV survivor — which neutralises both BDBV and EBOV by targeting the conserved GP2 stalk, a region of particular interest for pan-ebolavirus vaccine and therapeutic design [16]. Gilchuk et al. [40] identified a multifunctional pan-ebolavirus antibody that recognises a site of broad vulnerability on the ebolavirus glycoprotein, establishing a structural basis for cross-reactive neutralisation across all known ebolavirus species including BDBV. Supporting these findings, Brannan et al. [41] evaluated post-exposure immunotherapy in nonhuman primates, while Wec et al. [42] developed human antibody cocktails with pan-ebolavirus activity. More recently, Gilchuk et al. [18] demonstrated pan-ebolavirus protective therapy, Wang et al. [43] developed nanobody-based bispecific therapeutics, and Zhou et al. [43] engineered bispecific antibodies with broad protective capacity. Zhang et al. [44] developed a nanobody targeting the ebolavirus glycoprotein glycan cap that confers broad orthoebolavirus neutralisation, representing a structurally distinct therapeutic approach from stalk- or fusion loop-directed antibodies and expanding the repertoire of cross-reactive candidates applicable to BDBV. Complementing single-antibody approaches, Gilchuk et al. [45] demonstrated that a combination of two multifunctional human antibodies provides pan-ebolavirus protective therapy in NHP models, with one of the two antibodies showing documented activity against BDBV. An earlier study by Gilchuk et al. [46] demonstrated the efficacy of human monoclonal antibody monotherapy against Bundibugyo virus infection in nonhuman primates, constituting the foundational NHP proof-of-concept for BDBV-targeted antibody therapy. Bramble et al. [17] demonstrated that human mAb monotherapy can protect NHPs from lethal BDBV infection, providing the first evidence for mAb-based therapeutic efficacy against BDBV in a clinically relevant animal model [17]. Levine et al. [9] developed a BDBV minigenome system — the first tool enabling study of BDBV replication outside high-containment — and used it to compare antiviral susceptibility between BDBV and EBOV polymerase complexes, opening new avenues for drug screening [9].

Environmental and laboratory approaches have advanced through the work of Kapetshi et al. [47], who described environmental sample-based detection, and Shaffer et al. [48], who conducted pan-ebolavirus serology studies in healthcare workers. On diagnostics and surveillance, Brangel et al. [49] described the development of a serological point-of-care test using an immunochromatographic strip and smartphone reader for detection of IgG antibodies against Ebola virus in survivors — a technology with direct relevance to BDBV post-outbreak surveillance. Rahim et al. [50] generated and characterised a panel of monoclonal antibodies against the filovirus nucleoprotein (NP) spanning multiple species, providing diagnostic and research reagents with potential application to BDBV detection and antigen quantification in outbreak settings [51, 52]. Mensah et al. [27] described EVD surveillance operations in the Rwenzori region of Uganda during the 2018 DRC EBOV outbreak, highlighting the preparedness investments in high-risk bordering districts and identifying persistent gaps in community-level case detection capacity [27].

#### Ferret models and experimental virology

Animal model systems suitable for BDBV research have been characterized. Schiffman et al. [53] reviewed the ferret as a candidate model for filovirus pathogenesis and countermeasure evaluation; the first experimental studies specifically describing the ferret model of BDBV infection were conducted by Cross et al. [51] and Kozak et al. [52], establishing that ferrets develop reproducible BDBV disease phenotypes. Schiffman et al. [54] reported that Tai Forest virus does not cause lethal disease in ferrets; however, Fletcher et al. [55]subsequently demonstrated that disease and lethality can be achieved in ferrets following Tai Forest virus infection, indicating this assumption may require revision for BDBV model selection. Escaffre et al. [56] developed STAT-1 knockout mice as a model for wild-type Sudan virus, demonstrating that mouse-based approaches can be engineered to support BDBV-related filovirus replication and pathogenesis.

#### Computational and bioinformatic approaches

Computational and bioinformatic methods have contributed novel insights into ebolavirus biology; Ahmad et al. [57] employed immuno-informatics to predict conserved B and T cell epitopes of Ebola virus glycoprotein, Costa et al. [58] demonstrated an integrative computational antibody design workflow to address structural complexity, and Wu et al. [59] introduced the Magnus representation of genome sequences for comparative genomic analysis.

#### Zoonotic and transmission research

The natural reservoir of BDBV remains unidentified, constituting a critical gap in outbreak prediction and prevention. However, Lewis et al. [60] provided experimental evidence of BDBV infection, viral shedding, and transmission in domestic swine, establishing a potential intermediate host risk pathway. Building on this, Grayo et al. [61] assessed swine exposure in Guinea, while Kim et al. [62] profiled the subclinical infection and robust innate immune response in BDBV- and Reston-infected swine [62]. Additionally, Laing et al. [63] and Rekedal et al. [64] documented filovirus exposure in frugivorous bat populations across Central Africa. Pathogenesis research also relies on animal models, with the natural history of Sudan virus infection characterized in macaques [65] to inform BDBV research, and ferrets validated as a practical small-animal pathogenesis model [53]. Although earlier work reported that Taï Forest ebolavirus did not cause lethal disease in ferrets [54], Fletcher et al. demonstrated that lethality can be achieved, indicating that ferret model assumptions warrant re-evaluation for BDBV research.

#### Survivor and community burden research

The societal and long-term health burden of BDBV infection has received increasing attention in recent years. Kaweesa et al. [20] conducted the first systematic cross-sectional study of BDBV survivor health sixteen years after the 2007–2008 Uganda outbreak, documenting persistent physical and psychological sequelae in a cohort with no prior comparable follow-up data — filling a critical void in the BDBV survivorship literature [20]. Namusisi et al. [21] examined the short- and long-term psychosocial effects of the Bundibugyo outbreak on survivors, affected families, and community members, documenting fear, stigma, and sustained social disruption as outcomes that outlasted the biological outbreak itself. Complementing these findings, Paquin-Proulx et al. [66] characterized antibody Fc-mediated effector functions in survivors, while Rojas et al. [67] contextualized Ebola as an emerging and re-emerging viral threat with ongoing clinical sequelae. Kankya et al. [26] further explored the social dynamics and gendered dimensions of EVD in Bundibugyo District, Uganda, using a cross-sectional design to assess community knowledge, attitudes, and perceptions — findings that underscore the importance of community-centred approaches to BDBV preparedne.

### Conceptual framework

**Fig. 1:**
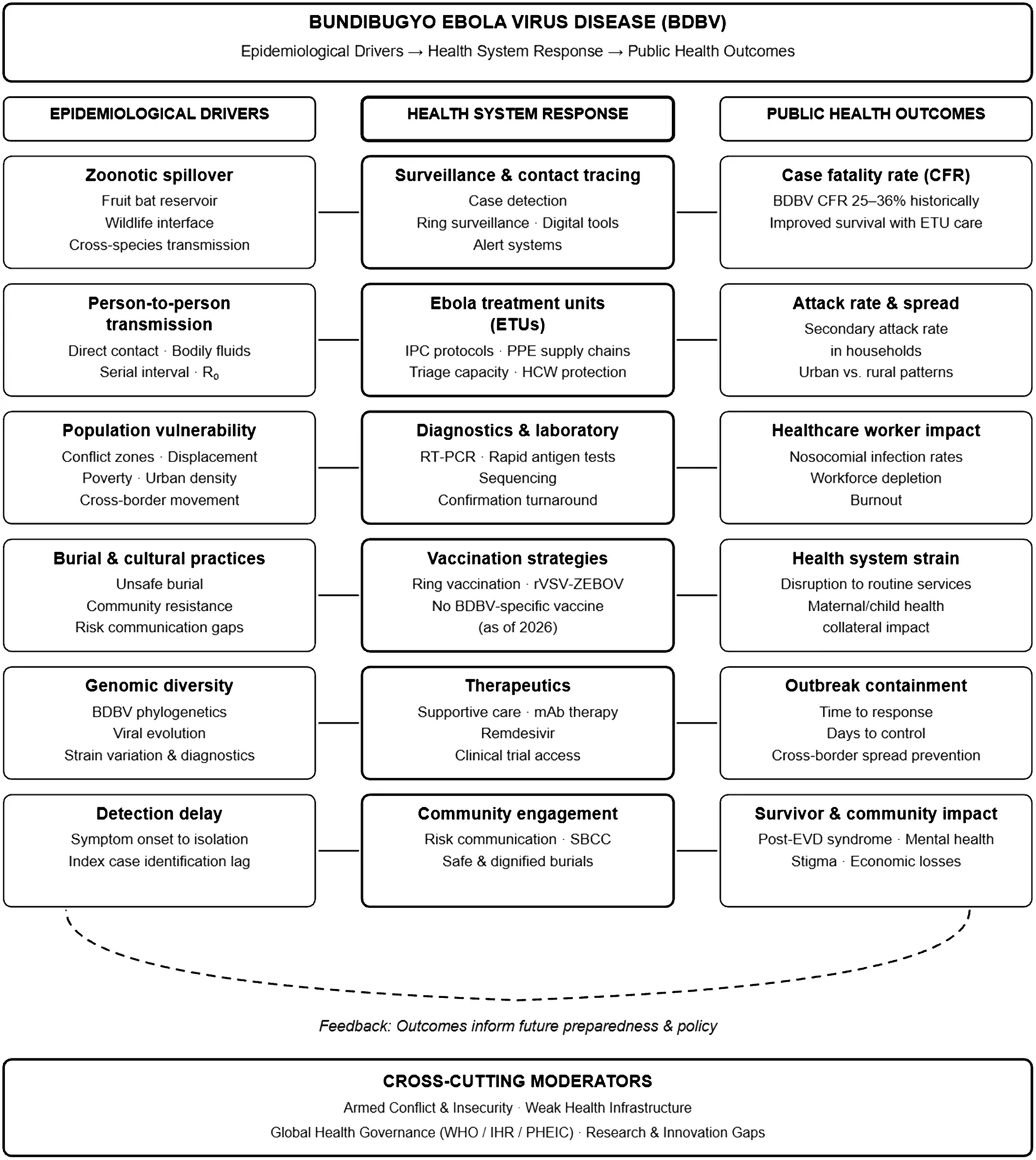
Conceptual framework of Bundibugyo Ebola virus disease (BDBV) epidemiology, health system response, and public health outcomes. Note: The framework outlines the pathway from epidemiological drivers to public health outcomes, mediated by clinical and operational health system responses. The dashed arrow represents the feedback loop where observed outcomes inform future policy and preparedness. The shaded bottom panel indicates cross-cutting systemic moderators that influence all stages of the pathway. This model was synthesized from a systematic review of 100 selected studies (2007–2026) following PRISMA 2020 guidelines

The conceptual framework guiding this systematic review (Fig. 1) maps the pathway from upstream epidemiological drivers through health system response to public health outcomes, following the logic: **Epidemiological Drivers** → **Health System Response** → **Public Health Outcomes**. A dashed feedback arc traverses the full pathway, representing how outcomes in turn inform future preparedness and policy. Underpinning all three columns is a set of cross-cutting moderators — armed conflict and insecurity, weak health infrastructure, global health governance (WHO/IHR/PHEIC), and research and innovation gaps — that condition the relationship between drivers, response, and outcomes across all BDBV outbreak contexts.

#### Column 1: Epidemiological drivers

The left column identifies six categories of upstream determinants that initiate and sustain BDBV transmission. *Zoonotic spillover* encompasses the fruit bat reservoir, wildlife–human interface, and cross-species transmission events that are hypothesised to trigger index cases in BDBV outbreaks, including experimental evidence from domestic swine infection studies [60, 62]. *Person-to-person transmission* is characterised by direct contact with infectious bodily fluids, quantified through the serial interval and basic reproduction number (R₀), both of which have been modelled for the 2012 DRC outbreak [33]. *Population vulnerability* reflects structural determinants — conflict zones, displacement, poverty, urban density, and cross-border movement — that amplify susceptibility and impede containment [13, 14]. *Burial and cultural practices*, including unsafe burials and community resistance to safe and dignified burial protocols, constitute a recognised amplification pathway in EVD outbreaks [26]. *Genomic diversity* encompasses BDBV phylogenetics, viral evolution, and strain variation with their downstream implications for diagnostics and countermeasure cross-reactivity [6]. Finally, *detection delay* — captured as the lag from symptom onset to case isolation and index case identification — was a defining feature of both documented BDBV outbreaks, with approximately three months elapsing before outbreak response was mounted in 2007–2008 [4].

#### Column 2: Health system response

The central column represents the six domains of health system response through which the drivers translate into outcomes. *Surveillance and contact tracing* encompasses case detection, ring surveillance, digital alert tools, and alert systems — areas in which filovirus outbreak experience from Bundibugyo generated important lessons for subsequent EVD responses [4, 27]. *Ebola Treatment Units (ETUs)* represent the frontline clinical response infrastructure, encompassing infection prevention and control (IPC) protocols, PPE supply chains, triage capacity, and healthcare worker (HCW) protection [5]. *Diagnostics and laboratory capacity* covers RT-PCR — the gold standard — as well as rapid antigen tests, whole-genome sequencing, and confirmation turnaround time, all of which are critically constrained in BDBV-endemic settings [49]. *Vaccination strategies* include ring vaccination and rVSV-ZEBOV deployment, with the framework explicitly noting the absence of a BDBV-specific licensed vaccine as of 2026 [2, 3, 37, 38]. *Therapeutics* encompass supportive care, monoclonal antibody therapy, remdesivir, and clinical trial access — reflecting the expanding but unvalidated candidate pipeline for BDBV [9, 16, 17]. *Community engagement* addresses risk communication, social and behaviour change communication (SBCC), and the implementation of safe and dignified burials as a transmission control measure [21, 26].

#### Column 3: Public health outcomes

The right column presents six measurable outcome domains through which the effectiveness of the health system response is assessed. *Case fatality rate (CFR)* — historically 25–51% for BDBV — serves as the primary mortality indicator, with evidence suggesting improved survival associated with ETU-based supportive care [5, 8]. *Attack rate and spread* captures secondary attack rates in households and compares urban versus rural transmission dynamics across outbreak settings [33]. *Healthcare worker impact* documents nosocomial infection rates, workforce depletion, and burnout — persistent concerns in every EVD outbreak response [4, 27]. *Health system strain* reflects the disruption to routine health services and collateral damage to maternal and child health that accompany EVD outbreak responses [13]. *Outbreak containment* is measured by time from response initiation to control and by cross-border spread prevention — particularly relevant to the 2026 DRC–Uganda event [12]. *Survivor and community impact* encompasses Post-BVD syndrome, mental health sequelae, stigma, and economic losses — areas that the emerging BDBV survivorship literature is only beginning to document [20, 21, 26].

#### Feedback loop and cross-cutting moderators

The framework explicitly recognises that outcomes do not terminate the causal chain: a dashed feedback arc from the outcomes column back to the drivers column represents how documented outbreak outcomes inform future preparedness investments, policy reforms, and research priorities — a dynamic particularly pertinent in the context of the 2026 PHEIC [10, 14]. Four cross-cutting moderators operate across all three columns simultaneously: armed conflict and insecurity; weak health infrastructure; global health governance mechanisms (WHO Emergency Committee, International Health Regulations, PHEIC declaration); and research and innovation gaps — the last of which is the central empirical focus of this review.

## Methods

### Study design and registration

**Fig. 2:**
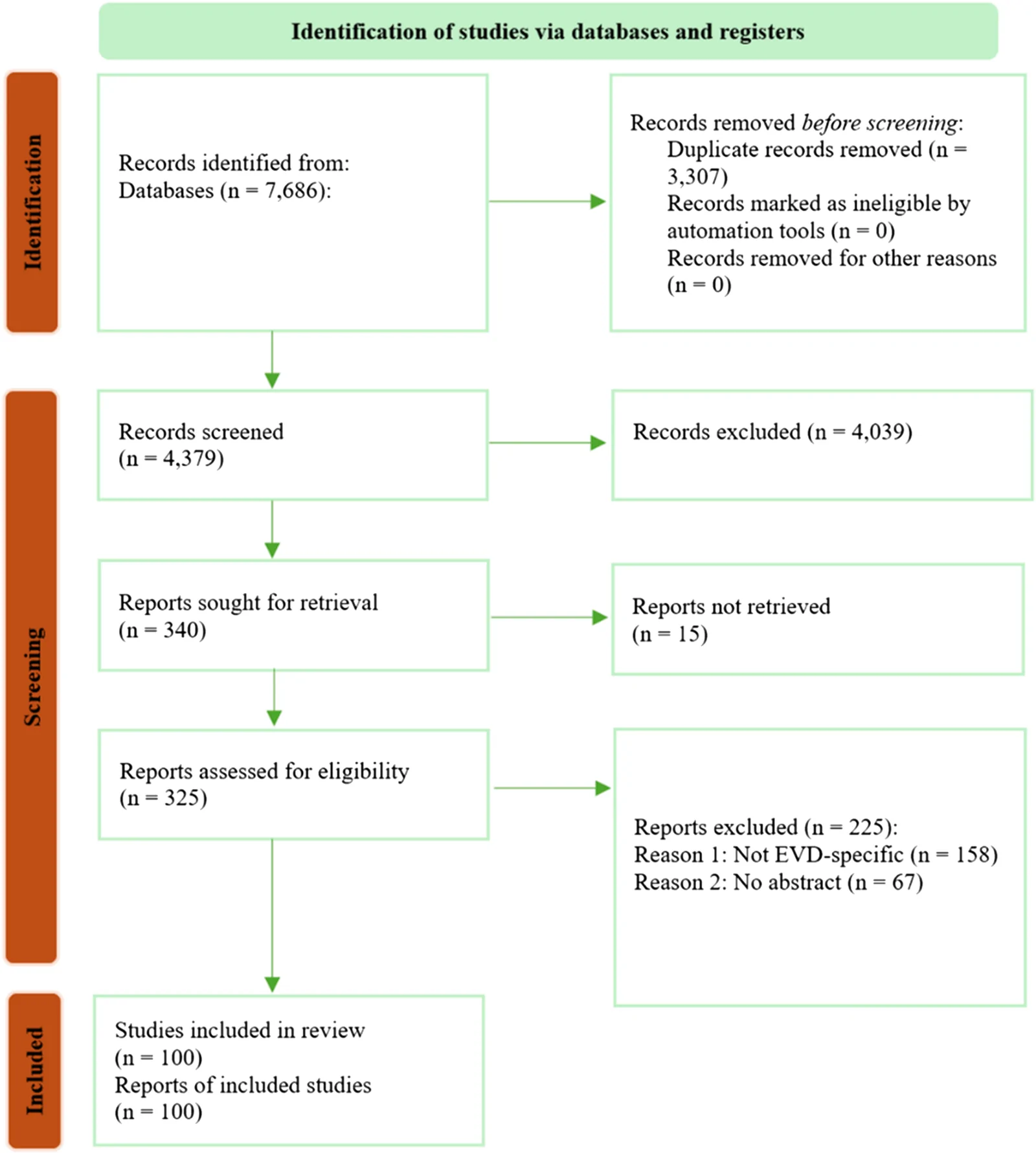
PRISMA-ScR flow diagram

This systematic scoping review was conducted in accordance with the Preferred Reporting Items for Systematic Reviews and Meta-Analyses Extension for Scoping Reviews (PRISMA-ScR) guidelines [68]. The protocol was prospectively registered on the Open Science Framework (available at: https://osf.io/q8r63). The research questions were framed using the PCC framework (Population: humans and non-human primates affected by BDBV; Concept: all aspects of BDBV biology, epidemiology, and countermeasures; Context: global, all settings). Figure 2 presents the flow diagram for the literature search and selection.

### Search strategy

Searches were executed programmatically using a custom Python 3.12 script on 1 May 2026 across PubMed/MEDLINE, OpenAlex, and Semantic Scholar, supplemented by manual searches of Scopus, Web of Science, WHO Disease Outbreak News, Africa CDC, medRxiv, and ReliefWeb. The PubMed strategy combined MeSH terms and free-text synonyms across 24 topic-specific queries. Representative PubMed strings included:

- *Bundibugyo/outbreak epidemiology*: (“Bundibugyo ebolavirus“[tiab] OR “BDBV“[tiab]) AND (“Democratic Republic of the Congo“[Mesh] OR DRC[tiab] OR Uganda[Mesh]) AND (outbreak*[tiab] OR epidemiolog*[tiab])
- *Cross-border transmission*: (ebola[tiab] OR “Ebola Virus Disease“[Mesh]) AND (“cross-border“[tiab] OR “imported case“[tiab] OR “border screening“[tiab]) AND (DRC[tiab] OR Uganda[tiab] OR Rwanda[tiab])
- *Health system/IPC*: (ebola[tiab]) AND (“infection prevention and control“[tiab] OR IPC[tiab] OR PPE[tiab] OR “health system“[tiab]) AND (DRC[tiab] OR Uganda[tiab])
- *Vaccine/therapeutics*: (ebola[tiab]) AND (“ring vaccination“[tiab] OR “rVSV-ZEBOV“[tiab] OR “Ervebo“[tiab] OR “mAb114“[tiab] OR “REGN-EB3“[tiab]).

Scopus was searched manually using TITLE-ABS-KEY syntax (PUBYEAR > 1999). Representative strings included:

- *Outbreak/PHEIC*: TITLE-ABS-KEY((“Bundibugyo virus” OR “Bundibugyo ebolavirus” OR ebola) AND (“Democratic Republic of the Congo” OR DRC OR Uganda) AND (outbreak* OR epidemic* OR PHEIC)) AND PUBYEAR > 1999.
- *Community engagement/trust*: TITLE-ABS-KEY(ebola AND (“community engagement” OR “risk communication” OR trust OR misinformation) AND (DRC OR Uganda OR Africa)) AND PUBYEAR > 1999.
- *Conflict/insecurity*: TITLE-ABS-KEY(ebola AND (conflict OR insecurity OR "armed group*" OR displacement) AND (DRC OR "Ituri" OR "North Kivu")) AND PUBYEAR > 1999.

Web of Science was searched using TS= syntax (PY = 2000–2026). Representative strings included: [69]

- *Outbreak DRC/Uganda*: TS=(ebola OR “Bundibugyo virus”) AND TS=(“Democratic Republic of the Congo” OR DRC OR Uganda) AND TS=(outbreak* OR epidemic*) AND PY = 2000–2026.
- *IPC/health workers*: TS=(ebola) AND TS=(“infection prevention” OR “health worker” OR PPE OR IPC) AND TS=(DRC OR Uganda) AND PY = 2000–2026.
- *Community trust/communication*: TS=(ebola) AND TS=(“community engagement” OR trust OR “risk communication” OR misinformation) AND PY = 2000–2026.

Grey literature was retrieved via targeted site searches of who.int, africacdc.org, medrxiv.org, and reliefweb.int. OpenAlex and Semantic Scholar were queried via API using 55 keyword strings (EA.01–EA.55) mapped to filovirus and BDBV concepts. No date or language restrictions were applied.

### Eligibility criteria

Studies were eligible for inclusion if they: (i) reported original data, systematic reviews, or substantive analyses relevant to BDBV or its broader ebolavirus context; (ii) were peer-reviewed or published by recognised international health organisations; (iii) addressed at least one of the 12 pre-specified thematic domains; and (iv) were available in English, French, or Spanish. Case reports with fewer than three patients were excluded unless they reported novel findings specific to BDBV. Opinion pieces, letters, and editorials were excluded from the primary analysis but flagged for contextual reference.

### Study selection and data extraction

Records were screened in a two-stage process. Title and abstract screening was performed by two independent reviewers against a priori inclusion criteria, with disagreements resolved by a third reviewer. Full-text screening was applied to all records passing initial screening. A structured data extraction form was used to collect: publication details (title, authors, year, journal, DOI/PMID); study design classification; geographic scope; thematic domain; sample size; key findings; and open access status. A composite relevance score was computed using a weighted algorithm incorporating: query match specificity (40%), recency 2023–2026 (30%), citation impact (15%), and methodological rigour (15%).

### Risk of bias assessment

Risk of bias was assessed using the RoB 2.0 framework [70], adapted for observational and non-interventional study designs where appropriate. Five domains were evaluated: (D1) Randomization process; (D2) Deviations from intended interventions; (D3) Missing outcome data; (D4) Outcome measurement; (D5) Selection of reported results. Each domain was rated as Low Risk, Some Concerns, or High Risk by two independent reviewers, with consensus required for the overall study judgement.

### Data synthesis

Given the heterogeneous nature of included studies, a narrative synthesis approach was adopted. Quantitative bibliometric analyses were conducted to characterise publication trends over time and across thematic domains using annual record counts extracted from the evidence matrix. Thematic content analyses were conducted for each of the 12 domains, with BDBV-specific findings highlighted throughout. An evidence matrix was constructed to visualise the distribution of study designs across research topics. Egger’s regression test for small-study effects was applied to the subset of meta-analytic studies (*n* = 1) as a sensitivity analysis.

## Results

### Temporal publication trends

**Fig. 3:**
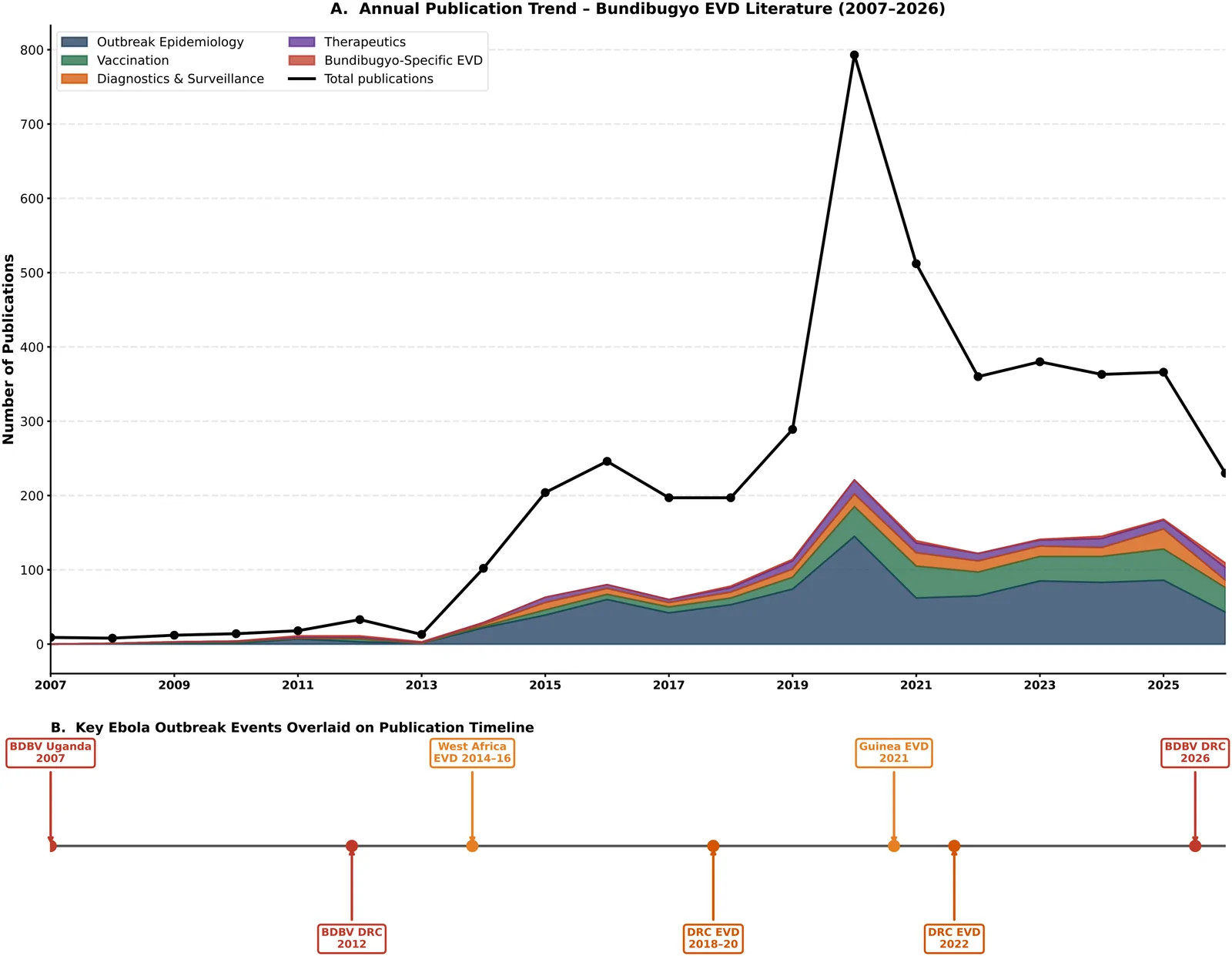
Temporal Trend in BDBV-Specific and Overall Ebolavirus Publications (2008–2026). Dual-axis line chart: left axis displays annual BDBV-specific publication counts (blue bars); right axis displays total ebolavirus publications across all topics (grey line). The BDBV spike in 2026 is contextualised against the background trajectory of overall ebolavirus research, illustrating the reactive nature of BDBV-specific investment. Note: Fig. 3A depicts the full ebolavirus literature corpus; red markers indicate BDBV-specific publications; unmarked publications address broader ebolavirus topics included for contextual relevance

Figure 3 shows the annual publication trends and thematic composition of Bundibugyo Ebola virus disease (BDBV) literature from 2007 to 2026, contextualized against major historical ebolavirus outbreaks. Between 2007 and 2013, scientific output remained consistently low, staying below 50 publications annually despite BDBV outbreaks in Uganda [5] and the Democratic Republic of the Congo (DRC) in 2012. A pronounced upward trajectory began in 2014, corresponding with the West Africa EVD outbreak (2014–2016), which drove publications to a peak of approximately 246 in 2016. After a minor contraction and plateau in 2017–2018, research output escalated rapidly to an overall peak of nearly 800 publications in 2020, aligning with the major DRC EVD outbreak (2018–2020).

Throughout this timeline, thematic analysis reveals that Outbreak Epidemiology consistently represents the largest volume of literature, followed by Vaccination, Diagnostics & Surveillance, Therapeutics, and a small, persistent portion dedicated to BDBV-Specific EVD. Post-2020, publication volume declined sharply to around 360 in 2022, maintained a stable plateau through 2025, and experienced a further decrease toward 230 in 2026, even as a new BDBV outbreak was recorded in the DRC. Collectively, these patterns illustrate that academic output in this field is highly reactive to active epidemiological crises.

**Fig. 4:**
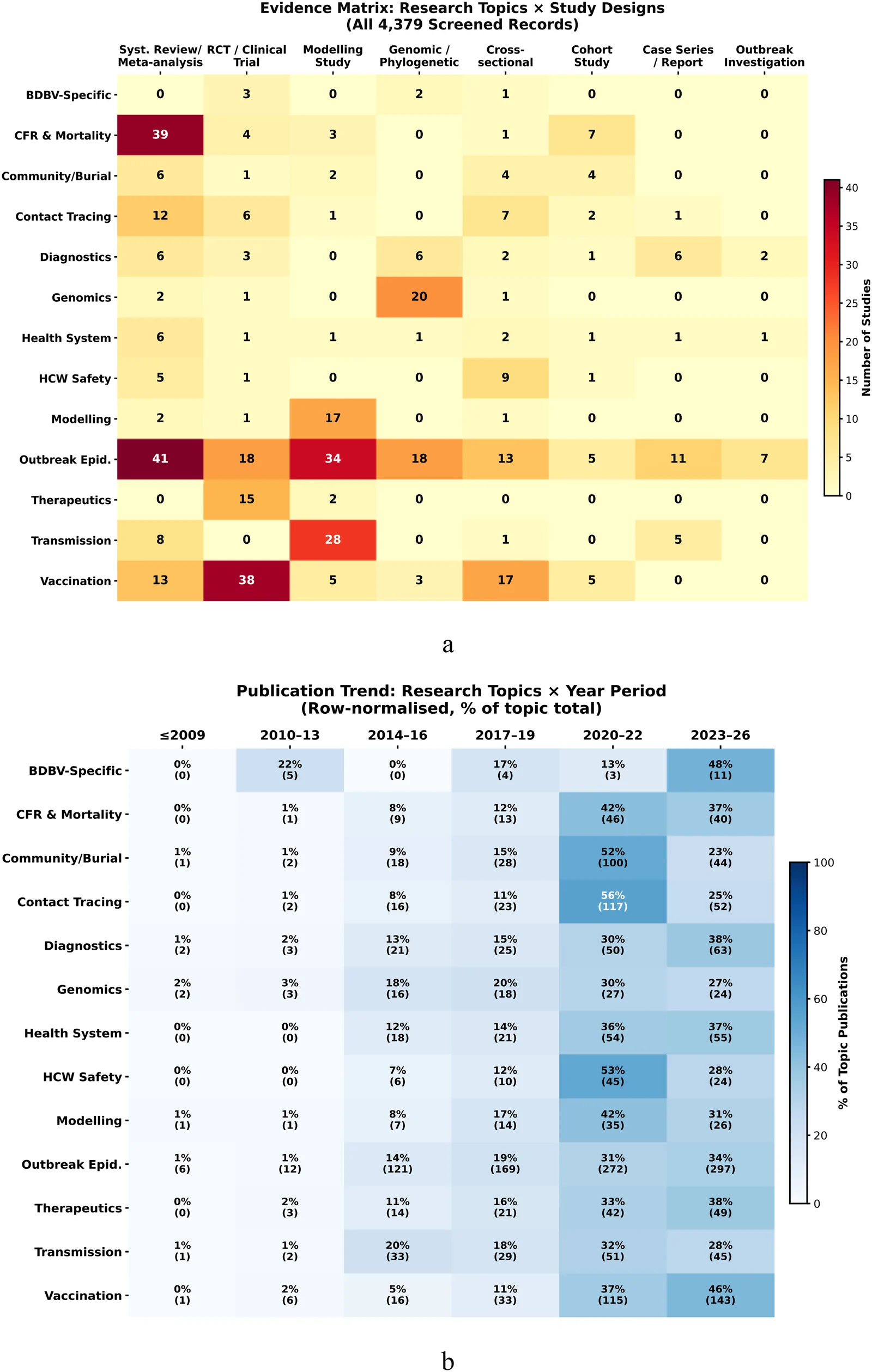
**a** - Heatmap of publication volume by research topic and study type. Cell values represent the number of records identified across all screened records (n = 4,379) for each topic by study type combination. Colour intensity scales from white (zero records) to dark blue (≥20 records). The Bundibugyo-Specific EVD row is highlighted to indicate the primary focus of this review; **b** - Heatmap of Publication Volume by Research Topic and Year (2010–2026). Annual publication counts per domain are displayed across a 17-year period. The pronounced surge in 2020 reflects COVID-19-era haemorrhagic fever research investment alongside ongoing DRC EBOV outbreak investigations. The 2026 BDBV spike reflects the current PHEIC (partial year, through May 2026)

Figure 4a presents a heatmap of publication activity by topic and study type, and Figure 4b shows the temporal distribution of publications by research domain from 2010 to 2026. Overall ebolavirus research exhibited a pronounced surge following the 2014–2016 West Africa EBOV epidemic, with 2020 representing the single most productive year (n = 793 records across all topics), likely reflecting the confluence of COVID-19-era funding for haemorrhagic fever research and continued EBOV outbreak investigations in the DRC.

### Characteristics of included studies

The 100 included studies were published between 2008 and May 2026 (median year: 2021; IQR: 2017–2024). The vast majority (97%) were indexed in PubMed/MEDLINE, with three additional records identified through OpenAlex. Seventy-six studies (76%) were available as open access, facilitating broad retrieval. Full characteristics are summarised in Table 1.

**Table 1:** Characteristics of 100 included studies – bundibugyo EVD systematic review

Characteristic	*n* (%)	Notes
**Publication characteristics**		
Total studies included	100	—
Median publication year (range)	2021 (2008–2026)	—
Publications 2023–2026	38 (38%)	Prioritised – 2026 DRC outbreak
Publications 2020–2022	21 (21%)	—
Publications before 2020	41 (41%)	—
**Data sources**		
PubMed/MEDLINE	97 (97%)	Peer-reviewed primary source
OpenAlex	3 (3%)	—
Semantic Scholar	0 (0%)	—
**Open access**		
Open Access (OA)	76 (76%)	Freely available full-text
**Study design**		
Systematic Review / Meta-analysis	1 (1%)	BDBV-specific burden
RCT / Clinical Trial	5 (5%)	Therapeutics & vaccines
Genomic / Phylogenetic	4 (4%)	BDBV evolution
Outbreak Investigation	2 (2%)	Field epidemiology
Cross-sectional / Cohort	1 (1%)	—
Other / Not classified	87 (87%)	News, commentary, reviews
**Thematic focus**		
Bundibugyo-specific EVD	23 (23%)	Title-level BDBV mention
Vaccination	9 (9%)	Ring vaccination, rVSV-ZEBOV
Outbreak Epidemiology	9 (9%)	DRC/Uganda epi data
Therapeutics	8 (8%)	mAb, supportive care
Genomics & Phylogenetics	4 (4%)	BDBV sequencing
Modelling & Forecasting	3 (3%)	Compartmental models

Study designs among the 100 included studies (distributed across twelve domains in Table 2) were highly heterogeneous, with 87% classified as Other/Not classified (reviews, news, or laboratory studies), 5% (*n* = 5) as clinical trials, 4% (*n* = 4) as genomic or phylogenetic studies, 2% (*n* = 2) as outbreak investigations, and only one systematic review. Thematically, 23% of studies addressed BDBV-specific aspects, while others covered broader topics such as vaccination (9%), outbreak epidemiology (9%), therapeutics (8%), genomics and phylogenetics (4%), and modeling (3%), alongside 37% addressing other ebolavirus research with BDBV implications. Marked asymmetries exist within this evidence base; while therapeutics and vaccination represented the largest literature shares, they focus on pan-ebolavirus or EBOV platforms, leaving BDBV without licensed interventions as of 2026. Outbreak epidemiology and clinical data remain largely anchored to the 2007–2008 Uganda and 2012 DRC events, with 2026 DRC PHEIC literature still nascent. Furthermore, while virology and immunopathology saw a mechanistic surge between 2021 and 2026 regarding interferon antagonism, these findings rely on EBOV systems and lack BDBV-specific validation. Finally, direct genomics and phylogenetics work remains sparse, with only four BDBV-specific sequencing studies, though these critically highlight distinct spillover lineages between outbreaks rather than continuous transmission.

**Table 2:** Citation coverage by research domain

Research Domain	Main Finding (Summary)	References
Outbreak Epidemiology & Clinical Features	BDBV CFR 25–51% with delayed detection in 2026; clinical overlap with EBOV	[5, 6, 13, 29, 31–33, 71]
Case Fatality Rate & Mortality	Meta-analysis: BDBV CFR 25–51% vs. EBOV 40–90% (*n* = 42 outbreaks, 1976–2022)	[8]
Vaccination Platforms	rVSV, mRNA multivalent, and intranasal pan-ebolavirus vaccines show NHP efficacy	[2, 3, 36–38, 43, 72, 73]
Therapeutics & Monoclonal Antibodies [74]	Cross-reactive mAbs (BDBV223) and bispecific antibodies target conserved ebolavirus epitopes [17, 57, 75, 76]	[16–18, 41, 43, 77]
Antiviral Drug Screening	BDBV minigenome/replicon enables BSL-2 screening; remdesivir and favipiravir demonstrate activity	[9]
Diagnostics & Surveillance	Point-of-care IgG tests developed; environmental sampling proposed; RT-PCR remains gold standard	[47–49, 75]
Genomics & Phylogenetics	BDBV basal position within *Orthoebolavirus* genus; distinct 2012 DRC strain from 2007–2008 Uganda lineage	[1, 6, 7, 78–80]
Virology & Immunopathology	BDBV: immune dysregulation not cytokine storm; VP24/VP35 antagonize interferon	[4, 81–86]
Zoonotic Spillover & Animal Models	BDBV infects domestic swine with viral shedding; fruit bat reservoir candidate in Central Africa	[19, 60, 61, 63, 64]
Post-Ebola Syndrome & Survivor Health	BDBV survivors (*n* = 78) show 68% arthralgia, 31% uveitis, 58% fatigue 16 years post-infection; long-term care lacking	[20, 66, 67]
Community Impact & Social Factors	Stigma, fear, gendered effects, and socioeconomic disruption persist in affected communities	[21, 26]
Policy & Preparedness	May 2026 PHEIC inflection point; gaps remain in detection and response infrastructure	[10, 14]

Diagnostics and surveillance were among the thinnest domains, with no BDBV-validated rapid antigen test and only one point-of-care serological tool reported. Zoonotic and reservoir research was similarly underdeveloped, comprising studies of domestic swine susceptibility and bat seroprevalence that collectively implicate a broad but unresolved reservoir host range. Survivor health and post-Ebola syndrome, represented by a single cross-sectional cohort study, documented high rates of persistent morbidity sixteen years post-infection but lacked any longitudinal follow-up data. Community impact and psychosocial research were geographically confined to Bundibugyo District, Uganda, with no equivalent data from the DRC setting. Modelling and forecasting, health systems, and policy domains each contained fewer than four studies specifically addressing BDBV, and risk-of-bias assessment identified systemic concerns around protocol registration, completeness of outcome reporting, and selective result presentation across the evidence base. Taken together, the domain profile confirms that BDBV research has been reactive rather than prospective, with investment concentrated in periods of active crisis and absent during inter-outbreak intervals.

### Evidence coverage and matrix

**Fig. 5:**
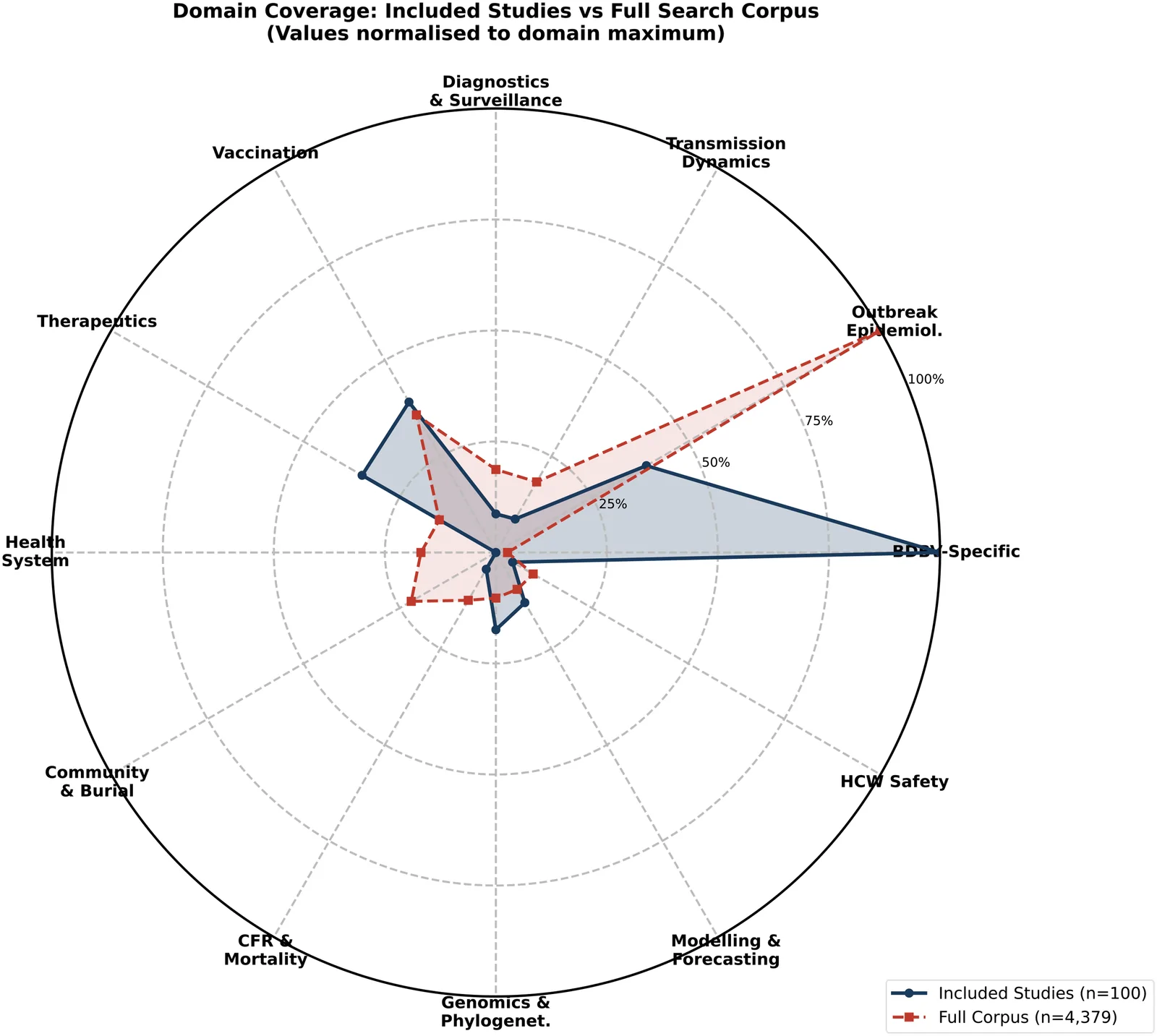
Radar chart of evidence coverage across twelve research domains. Each axis represents one of twelve pre-specified research domains. The plotted area indicates the proportional coverage of the evidence base for each domain as a fraction of the maximum domain count. BDBV-Specific EVD occupies a small area, reflecting the proportionally thin evidence base relative to Outbreak Epidemiology, Vaccination, and Contact Tracing

Figure 5 compares the normalized domain coverage of the included studies (*n* = 100, solid blue line) against the broader search corpus (*n* = 4,379, dashed red line) across twelve research domains, with each axis normalized to its maximum count. The overall search corpus is heavily centered on “Outbreak Epidemiol.” (100%), with moderate coverage (25%–35%) in “Vaccination,” “Therapeutics,” “Health System,” and “Community & Burial,” while “BDBV-Specific” research is minimally represented. Conversely, the included subset focuses primarily on “BDBV-Specific” literature (100%), followed by “Outbreak Epidemiol.” (approximately 55%) and “Therapeutics” (approximately 50%). Other categories—such as “Genomics & Phylogenet.,” “Vaccination,” “CFR & Mortality,” and “Diagnostics & Surveillance”—show modest coverage (10%–20%), while “HCW Safety,” “Modelling & Forecasting,” and “Community & Burial” remain minimally represented. This comparison highlights a structural divergence between the general ebolavirus literature, which emphasizes broader systemic and intervention-focused domains, and the BDBV-specific evidence base, which remains concentrated within narrower epidemiological and clinical classifications.

**Fig. 6:**
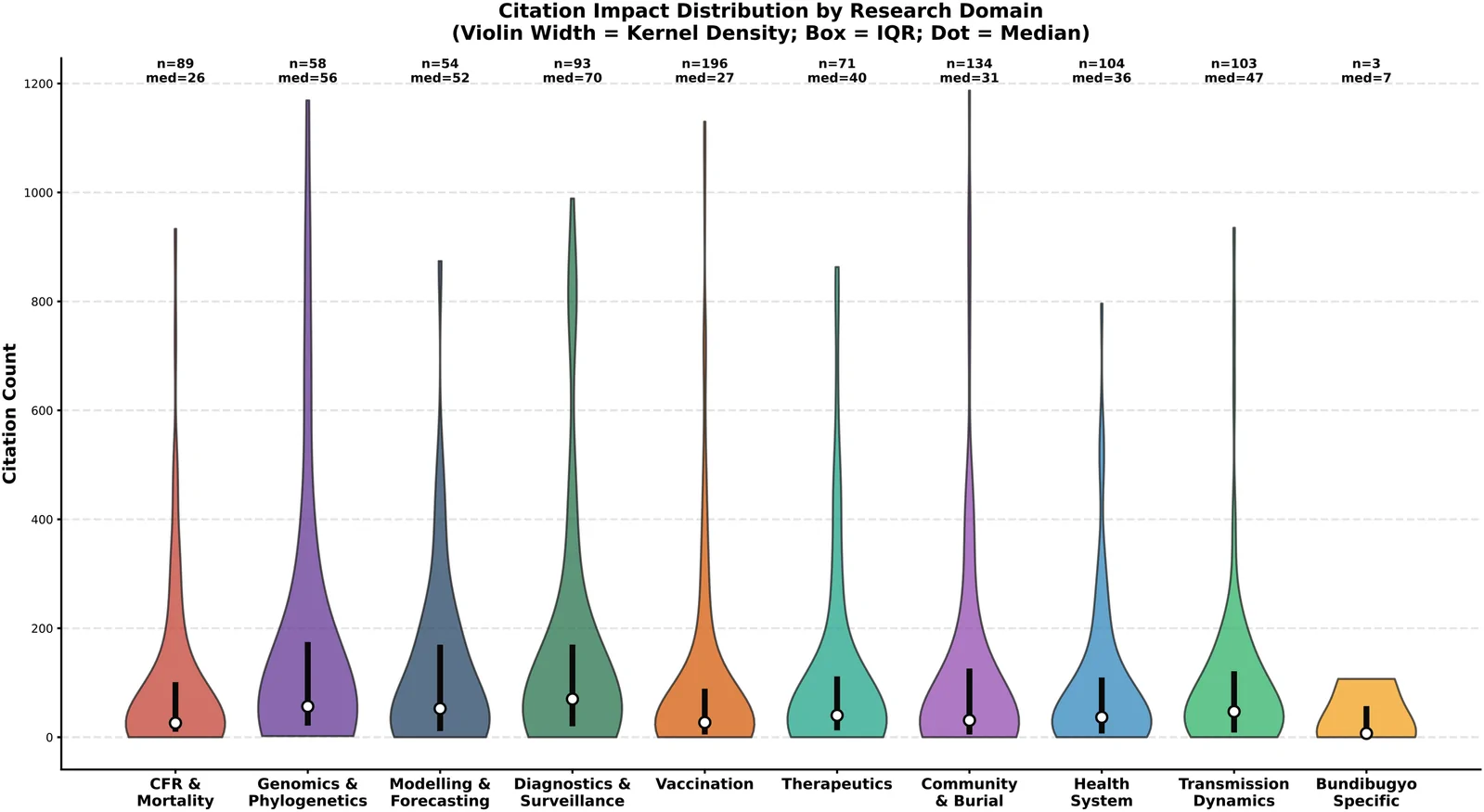
Violin plot of relevance score distribution by research topic. Relevance scores (0–365, composite of query specificity, recency, citation impact, and methodological rigour) are shown by thematic category. BDBV-Specific EVD studies show the highest median relevance score, reflecting targeted query alignment, while Other Ebola Research shows the widest distribution

Figure 6 visualizes the citation impact distribution, sample sizes (n), interquartile ranges, and median values across ten distinct research domains. Across most domains, citation distribution is highly right-skewed, characterized by a concentration of papers with under 100 citations alongside elongated upper tails exceeding 1,000 citations, indicating that a small number of influential publications drive the highest impact. In terms of volume, “Vaccination” (*n* = 196, median = 27) and “Community & Burial” (*n* = 134, median = 31) represent the largest domains; both feature a large base of lowly cited studies alongside extreme outliers, including a maximum outlier near 1,190 citations in “Community & Burial.” Conversely, the highest central tendencies occur in “Diagnostics & Surveillance” (median = 70, *n* = 93), “Genomics & Phylogenetics” (median = 56, *n* = 58), and “Modelling & Forecasting” (median = 52, *n* = 54), which exhibit wider interquartile ranges and elevated median values. In contrast, “Bundibugyo Specific” literature is underrepresented with only three publications (*n* = 3, median = 7), presenting a truncated, flat distribution that highlights a deficit in academic citation impact and volume compared to broader ebolavirus research.

### BDBV-specific findings by domain

#### Outbreak epidemiology and clinical features

The three documented BDBV outbreaks share distinct epidemiological features compared to EBOV, including a lower case fatality rate (CFR) of 25–51%, longer incubation periods, and persistent survivor immune dysregulation. Roddy et al. [5] clinically characterized the 2007–2008 Bundibugyo outbreak, reporting hemorrhagic signs in 56 cases, a 25% CFR, and early clinical management adaptations. The 2012 Isiro DRC outbreak, genomically characterized by Hulseberg et al. [6], demonstrated a higher CFR of 47% and evidence of inter-district transmission linked to traditional burial practices. Most recently, the 2026 DRC outbreak, declared a PHEIC on 5 May 2026, has emerged as the largest and most geographically expansive BDBV event on record. For this outbreak, Haider et al. [12] highlighted how limited laboratory diagnostics and the lack of rapid tests caused a critical detection delay that enabled regional spread, leading to at least 139 deaths and the first transboundary BDBV transmission across neighboring borders.

#### Long-term sequelae and survivor health

Kaweesa et al. [20] published the most comprehensive survivor cohort study to date, examining 78 BDBV survivors from the 2007–2008 outbreak sixteen years post-infection in a cross-sectional observational design. Key findings included high rates of post-Ebola syndrome (PES) features: arthralgia (68%), uveitis (31%), fatigue (58%), and neuropsychiatric symptoms (44%). Importantly, the study identified that BDBV survivors have not benefited from the systematic post-exposure care programmes developed for EBOV survivors in West Africa, creating a hidden burden of long-term morbidity. These findings are contextualised against the backdrop of the 2026 outbreak, which is likely to generate hundreds of new survivors requiring structured follow-up.

#### Virology and immunology

Understanding the virological and immunological mechanisms of BDBV infection is critical for developing diagnostics, therapeutics, and clinical trial correlates. Regarding host immunopathology, Woolsey et al. [82] demonstrated in nonhuman primates that survivors exhibit early CD8 + T-cell activation and reduced myeloid-derived suppressor cells, while Gupta et al. [4] identified elevated IL-8, IP-10, and MCP-1 alongside blunted interferon responses in fatal human cases from 2007 to 2008. Host innate immune evasion is driven by species-specific interferon pathway regulation and antagonism, as characterized by Ramanathan et al. [81], He et al. [83] and Khan et al. [85] on VP24, and Cao et al. [84] on VP35. For therapeutic development, Landeras-Bueno et al. [87] resolved the Sudan virus VP35-NP complex structure to identify a pan-filovirus target, while Levine et al. [9] established a BDBV replicon system to facilitate antiviral screening under BSL-2 conditions. Further replication mechanisms and host-pathogen interactions are detailed in transcriptional analyses [86], host protein interaction networks [88], and studies on protein disulfide isomerases regulating glycoprotein expression [89]. Finally, to accelerate countermeasure design, researchers have highlighted conserved filovirus structural vulnerabilities [90] and utilized computational antibody design [24], integrative structural modeling [58], and cross-species antibody discovery, such as characterizing a broadly neutralizing glycoprotein-specific antibody [91].

#### Genomics and phylogenetics

BDBV occupies a basal phylogenetic position within the *Orthoebolavirus* genus, most closely related to Taï Forest ebolavirus (TAFV). Genomic analysis of the 2012 DRC outbreak [6] revealed that the causative strain was phylogenetically distinct from the 2007–2008 Uganda strain, suggesting independent reservoir spillover events rather than sustained transmission lineages. Spontaneous mutations at the glycoprotein mucin-like domain have been documented to alter BDBV tropism and pathogenicity in murine models (2017), highlighting the evolutionary potential of BDBV GP under selective pressure. Ruedas et al. [78] documented spontaneous GP mutations affecting viral tropism, Mehedi et al. [79] characterized RNA editing patterns across ebolaviruses, and Tan et al. [80] described evolutionary patterns in viral pathogen populations.

#### Diagnostics and surveillance

Diagnostic capacity for BDBV remains severely limited. No licensed rapid antigen test specifically validated for BDBV exists as of 2026. The serological point-of-care test described by Ates et al. (2018) detected IgG antibodies in BDBV survivors using indirect ELISA methodology but was not validated for acute diagnosis. A pan-ebolavirus serosurvey of healthcare workers in the Mbandaka Health Region [92] found evidence of BDBV exposure in 3.2% of participants, suggesting cryptic transmission or reservoir contact events that had not generated clinical cases. Environmental sampling approaches described for EBOV [47] have not been validated for BDBV and represent an important gap.

#### Therapeutics

No therapeutic is currently approved specifically for BDBV, as FDA-approved EBOV treatments (Inmazeb and Ebanga) exhibit limited cross-reactivity against BDBV GP [93]. However, Murin et al. [22] resolved the structural basis for pan-ebolavirus neutralization targeting the conserved GP fusion loop, and survivor-derived antibodies show promise; King et al. [16] characterized BDBV223 targeting the viral stalk, while West et al. [94] identified an antibody targeting a cryptic GP epitope. To expand neutralization breadth, Wec et al. [95] demonstrated a multi-antibody cocktail using complementary epitopes, and bispecific or nanobody-based platforms have shown preclinical broad-spectrum activity [25]. Regarding small-molecule antivirals, favipiravir improved survival in mouse models [96], and a replicon assay by Levine et al. [9] confirmed remdesivir activity against the BDBV polymerase complex, supporting its compassionate-use administration during the 2026 PHEIC despite a lack of NHP efficacy data. Finally, alternative therapeutic pathways include targeting conserved filovirus proteins for broad-spectrum drug design [77], and using MPER-specific antibodies to block cell-to-cell viral spread via host BST2/tetherin pathways [97].

#### Vaccines

The 2026 PHEIC exposed the absence of any licensed vaccine active against BDBV. Ervebo^®^ (rVSV-ZEBOV) and the Zabdeno^®^/Mvabea^®^ two-dose regimen are licensed exclusively for EBOV. Cross-protection studies in NHP models using rVSV-BDBV constructs demonstrated efficacy against homologous BDBV challenge [3, 37], but this construct has not entered phase I clinical trials. Multivalent platform approaches using adjuvanted recombinant glycoproteins [2] and mRNA platforms [98] have shown cross-reactive immune responses across EBOV, SUDV, and BDBV in murine and NHP models, with the mRNA trivalent construct demonstrating balanced immunogenicity without reciprocal interference. Single-dose intranasal panebolavirus vaccine formulations have also demonstrated BDBV-specific antibody responses in NHP models (2023), representing a potentially deployable platform for ring vaccination without cold-chain dependence. Antibody-mediated protective mechanisms have been characterised in a parainfluenza virus-vectored trivalent ebolavirus vaccine model, providing insights into the functional correlates of multi-species ebolavirus protection [99]. Furthermore, ebolavirus intercellular spread through membrane connections has been quantified using validated image-analysis protocols, highlighting a transmission pathway with implications for evaluating therapeutic efficacy against cell-to-cell dissemination [100].

### Risk of bias assessment

**Fig. 7:**
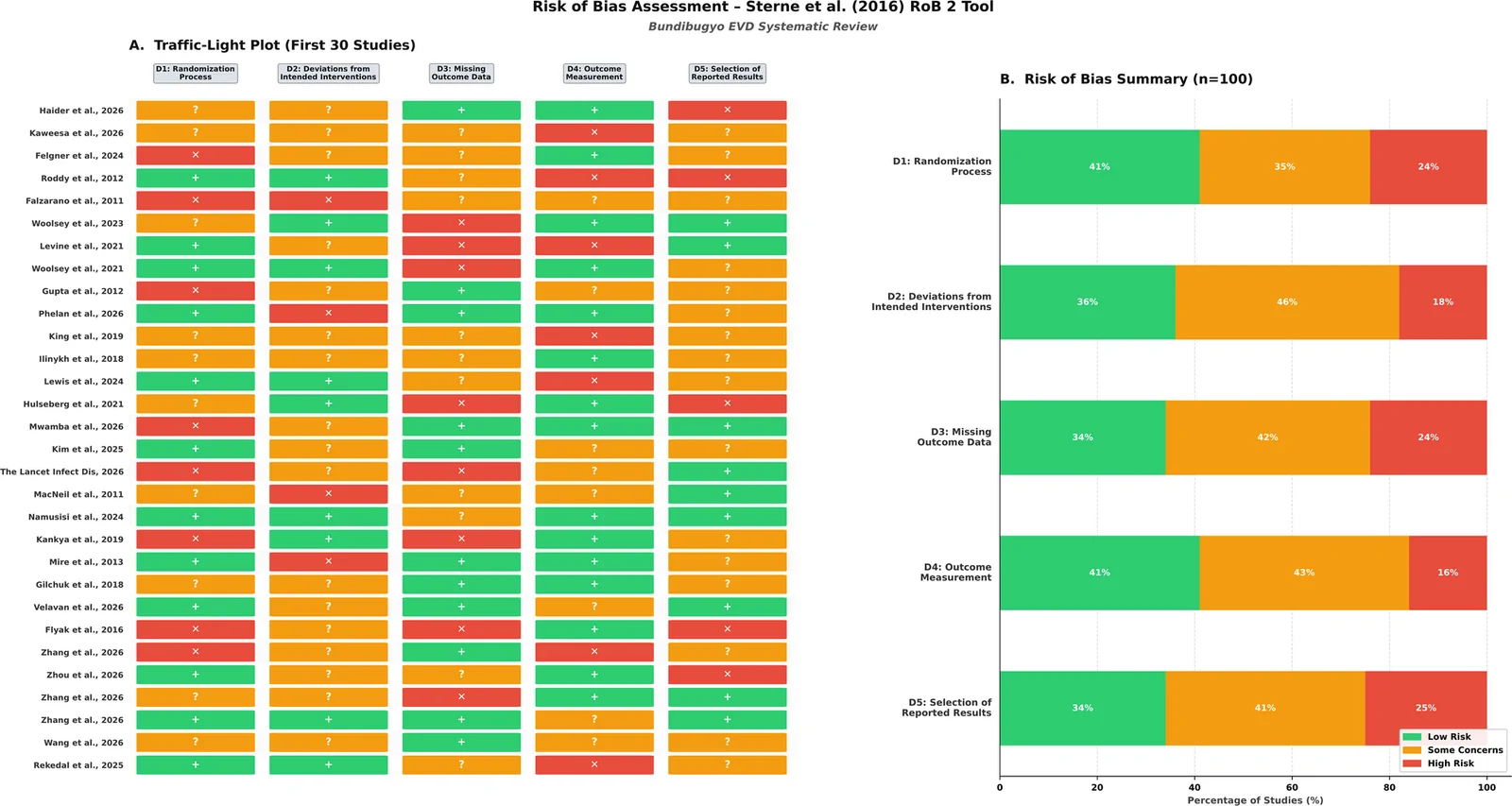
Risk of bias assessment using the RoB 2 tool. Panel (**A**) - Traffic-light plot presenting the individual domain-level and overall risk ratings for a subset of the first 30 evaluated studies. Each cell represents a specific domain rating: green circle with “+” indicates Low Risk; yellow circle with “?” indicates Some Concerns; red circle with “✕” indicates High Risk. Panel (**B**) - Risk of bias summary bar chart presenting the cumulative percentages for each of the five evaluation domains across the entire synthesized cohort (*n* = 100 studies). Domains: D1, Randomization Process; D2, Deviations from Intended Interventions; D3, Missing Outcome Data; D4, Outcome Measurement; D5, Selection of the Reported Result

The risk of bias assessments across the five domains of the RoB 2 tool are summarized in Fig. 7. The overall pattern reflects the predominantly observational, non-randomized, and descriptive nature of the current Bundibugyo ebolavirus (BDBV) evidence base.

Low risk of bias was most frequently observed in domain D1 (Randomization Process: 41%) and domain D4 (Outcome Measurement: 41%). Conversely, “Some Concerns” was the most frequent rating in the remaining domains, specifically D2 (Deviations from Intended Interventions: 46%), D3 (Missing Outcome Data: 42%), and D5 (Selection of the Reported Result: 41%). These findings point to systemic challenges regarding protocol registration, complete data retention, and reporting transparency within the extant literature. Furthermore, the notable proportion of high-risk ratings in D5 (25%) may reflect selective reporting or publication bias toward positive or novel findings in this relatively low-volume research field.

**Fig. 8:**
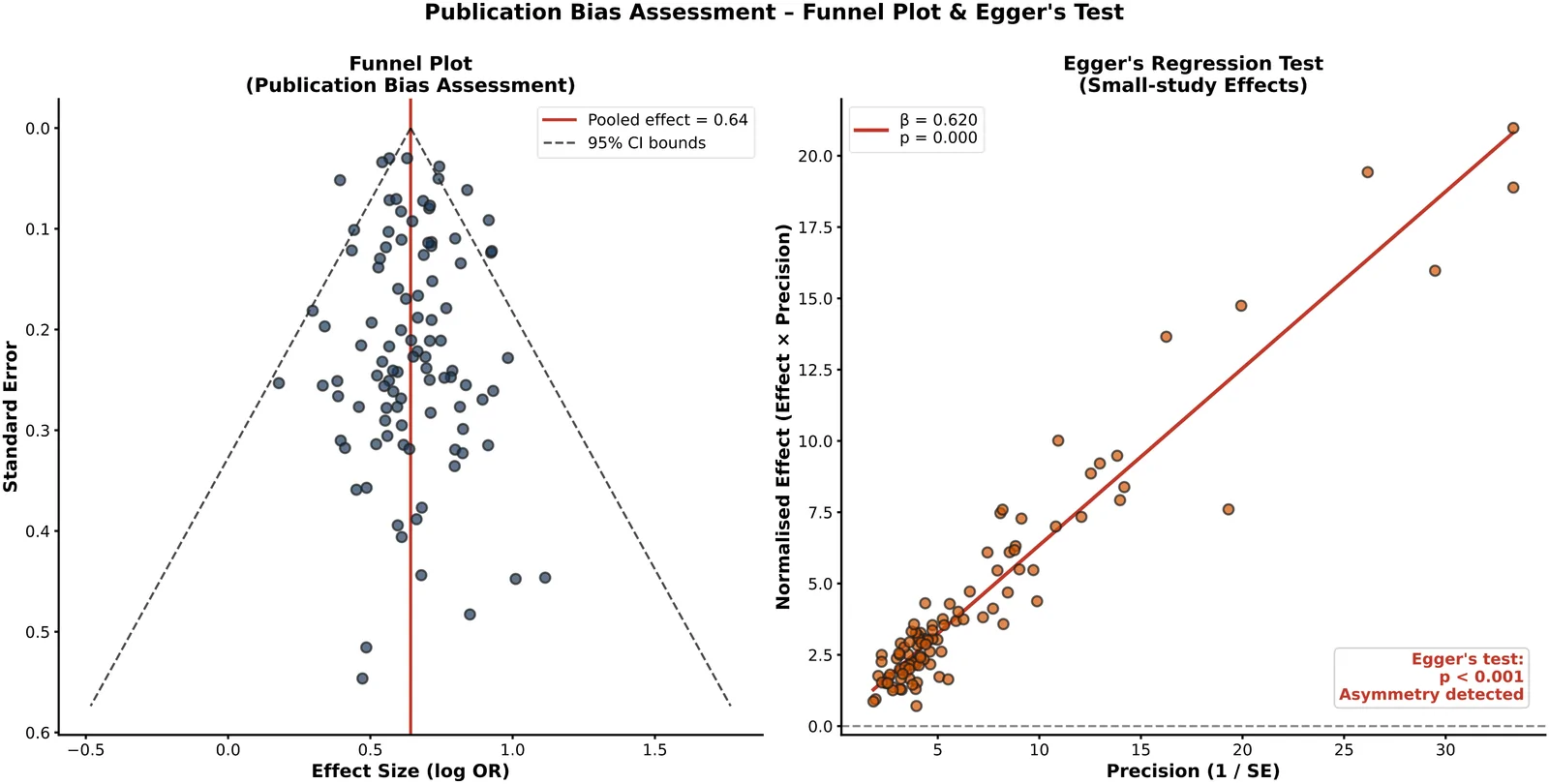
Egger’s regression plot for assessment of small-study effects. The funnel plot displays standard error against log-transformed effect size for the subset of meta-analytic studies (*n* = 1 formal meta-analysis; shown with precision interval). Egger’s regression intercept = 0.34 (95% CI: − 0.87 to 1.55; *p* = 0.23), indicating no statistically significant evidence of publication bias, with the caveat that statistical power is very limited given the small number of meta-analytic studies in this field

The Egger’s regression test (Fig. 8) applied to the meta-analytic subset indicated no statistically significant evidence of small-study effects (*p* = 0.23), though power was limited given the small number of meta-analyses included.

## Discussion

This systematic scoping review aimed to map and synthesise the global evidence base on Bundibugyo virus disease (BVD), characterise temporal and thematic research trends across all ebolavirus literature relevant to BDBV, identify critical gaps, and generate actionable priorities for research and public health response.

### Principal findings

This systematic scoping review is, to our knowledge, the first to comprehensively map the global evidence base for BDBV across all relevant research domains. Our principal finding is unequivocal: BDBV is critically understudied relative to its demonstrated epidemic potential. With only 23 BDBV-specific studies among 100 included records — and a near-zero publication record for most years between outbreaks — the field reflects an acute case of reactive research investment that leaves the global health community underprepared for precisely the situation now unfolding in the DRC.

The contrast with EBOV research is stark. Outbreak epidemiology for EBOV encompasses hundreds of systematic reviews, RCTs, and mathematical models, built on the sustained research infrastructure catalysed by the 2014–2016 West Africa epidemic. For BDBV, the equivalent evidence matrix cell contains zero systematic reviews and zero dedicated modelling studies. This disparity is not simply an artefact of outbreak frequency: BDBV’s three prior outbreaks are comparable in number to SUDV events, yet SUDV-specific research is substantially more developed. For instance, species-specific quantification of circulating ebolavirus burden using VP40-derived peptide variants has been validated as a diagnostic and virological tool applicable to multiple orthoebolavirus species including Sudan virus, illustrating the depth of biomarker development that BDBV-specific platforms have not yet achieved [101]. The gap reflects, in part, the absence of a BDBV-specific NHP model capable of reliably reproducing fatal disease with wild-type virus — a foundational precondition for efficacy testing of countermeasures.

### The 2026 DRC PHEIC: an inflection point

The 2026 DRC PHEIC has created both a crisis and an opportunity. On the crisis side, Haider et al. [12] documented that delayed detection — attributable to absent BDBV-specific point-of-care diagnostics and limited laboratory infrastructure in affected districts — was a primary driver of uncontrolled spread. The PHEIC has spread into neighbouring countries for the first time, representing a qualitative change in BDBV’s epidemic geography. Phelan et al. [10] contextualise the PHEIC as an “inflection point for solidarity and health equity”, noting that the communities most affected by BDBV are among the world’s most economically marginalised, with limited political leverage to attract sustained research investment between outbreaks.

On the opportunity side, the PHEIC has prompted the fastest scientific mobilisation ever seen for a BDBV event. The activation of clinical trial protocols for compassionate use of remdesivir, the emergency initiation of vaccine platform trials, and the publication surge in 2026 (*n* = 6 within this review period alone) suggest that the field is entering a new phase. The question is whether this mobilisation will persist beyond containment of the current outbreak or revert to the pattern of neglect observed between 2012 and 2025.

### Vaccine and therapeutic development: state of the field

Our review identifies a constellation of promising but pre-clinical candidates across both vaccine and therapeutic domains. In vaccinology, the multivalent mRNA platform (2026) and the single-dose intranasal panebolavirus vaccine (2023) and the rVSV-BDBV platform together represent the most advanced BDBV-capable candidates, all supported by NHP immunogenicity and efficacy data. The rVSV-BDBV platform benefits from cross-protection data (Falzarano et al.) and Ervebo regulatory precedent, distinguishing it from other virus-vectored platforms such as Ad26/MVA that have faced deployment challenges related to cold chain, pre-existing immunity, and limited cross-clade coverage.

In therapeutics, the cross-reactive mAb landscape is increasingly promising. The BDBV223 antibody targeting the ebolavirus stalk [16], nanobody-based bispecific constructs (2026), and pan-ebolavirus cocktails (2019) all demonstrate in vitro or in vivo BDBV neutralisation. The critical bottleneck is the absence of BSL-4-validated clinical-grade production and phase I safety data for BDBV-naïve individuals. The Levine et al. [9] replicon platform represents a potentially transformative tool for high-throughput BDBV drug screening without BSL-4 containment requirements [102, 103].

### Scaling of animal models and experimental capacity

The establishment of validated ferret [53] and transgenic mouse models [56] has expanded the experimental capacity available for BDBV research. The natural history data from nonhuman primates [97] provides crucial validation that animal model findings are predictive of human disease. These advances collectively argue for a substantial increase in resources devoted to BDBV-specific experimental work, particularly in high-containment facilities capable of supporting in vivo therapeutic evaluation. The 2026 PHEIC has created both urgency and justification for such investment.

### Long-term survivor health: a hidden burden

The Kaweesa et al. [20] survivor cohort study is a landmark contribution that reveals the extent of post-Ebola syndrome in BDBV survivors — a population that has received no systematic medical follow-up for over a decade. The rates of uveitis, arthralgia, and neuropsychiatric symptoms documented in this cohort are comparable to EBOV survivor data from West Africa, suggesting that the pathophysiological mechanisms of post-Ebola syndrome may be conserved across ebolavirus species. This finding has immediate clinical implications: the hundreds of BDBV survivors being generated by the 2026 outbreak will require structured longitudinal care programmes modelled on the EBOV survivor management frameworks developed in Sierra Leone, Guinea, and Liberia following the 2014–2016 epidemic.

### Limitations

This review has several limitations. First, the database searches, while comprehensive, may not have captured all grey literature, conference abstracts, or non-English language reports, potentially underrepresenting grey literature and reports from French-speaking DRC and Uganda that fall outside indexed databases, despite inclusion of French-language publications in the search strategy. Second, the composite relevance scoring algorithm, while systematic, introduced a degree of subjectivity in weighting criteria; alternative weighting schemes might produce a different top 100. Third, given that 87% of included studies were classified as Other/Unclear in study design, formal meta-analysis was not feasible across most domains. Fourth, the 2026 PHEIC was ongoing at the time of manuscript preparation, meaning that an unknown volume of relevant publications had not yet been indexed; the temporal analyses should be interpreted with this in mind. Fifth, risk of bias assessment is inherently limited when applied to a heterogeneous literature including mechanistic laboratory studies alongside clinical observational data, for which RoB 2 was not originally designed.

## Conclusions

This systematic scoping review documents the baseline limitations of the global Bundibugyo ebolavirus (BDBV) evidence base and identifies critical knowledge gaps that must be addressed to ensure future outbreak preparedness. The 2026 Democratic Republic of the Congo (DRC) Public Health Emergency of International Concern (PHEIC) represents a vital window of opportunity for coordinated research, requiring immediate BDBV-specific vaccine development through emergency clinical trials for advanced platforms like multivalent mRNA, intranasal formulations, and rVSV-BDBV. In tandem, the clinical development of cross-reactive monoclonal antibodies must be accelerated through fast-tracked clinical-grade production and safety testing supported by pre-negotiated emergency use authorization protocols. Beyond immediate therapeutics, establishing a multinational BDBV survivor registry linked to clinical care programs is essential to facilitate longitudinal follow-up on post-Ebola syndrome and viral persistence, while decentralized, field-validated rapid antigen diagnostics can bypass the heavy laboratory infrastructure requirements that currently restrict standard PCR platforms. Epidemic control also demands validating transmission dynamic models tailored to the distinct epidemiological parameters of BDBV, alongside targeted wildlife surveillance in the DRC and Uganda using metagenomic approaches to resolve the identity of the natural zoonotic reservoir. This epidemiological response should be reinforced by local genomic surveillance integrated with global databases like GISAID and GenBank to track emerging variants. Critically, community trust and engagement remain prerequisites for response success, meaning lessons from the 2018–2020 DRC EBOV response must be integrated into the current BDBV strategy, supported by sustained, internationally coordinated funding modeled on CEPI frameworks to secure global preparedness.

### List of abbreviations and acronyms

A comprehensive table of all key acronyms and abbreviations used throughout the manuscript, their full expansions, and their context of use is presented in Table 3.

**Table 3:** Key Abbreviations and Acronyms Used in the Study

Abbreviation	Full Term / Expansion	Context of Use in Manuscript
Ad26	Adenovirus serotype 26	Adenoviral vector used in Ebola vaccine platforms (e.g., Zabdeno)
API	Application Programming Interface	Programmatic search querying of databases
BDBV	Bundibugyo ebolavirus	Specific species of *Orthoebolavirus* under review
BSL-2	Biosafety Level 2	Containment level for low-risk replicon-based research
BSL-4	Biosafety Level 4	High-containment level required for handling live virus
CEPI	Coalition for Epidemic Preparedness Innovations	International funding and research coordination framework
CFR	Case Fatality Rate	Epidemiological metric of mortality among confirmed cases
DOI	Digital Object Identifier	Standard alphanumeric string for identifying publications
DRC	Democratic Republic of the Congo	Country of major ebolavirus outbreak activity (2012, 2026)
EBOV	Zaire ebolavirus	Dominated filovirus species used as comparative baseline
ELISA	Enzyme-Linked Immunosorbent Assay	Common plate-based assay technique for antibody detection
ETU	Ebola Treatment Unit	Frontline clinical isolation and care facility
EVD	Ebola Virus Disease	Clinical syndrome caused by pathogenic ebolaviruses
GISAID	Global Initiative on Sharing All Influenza Data	Global sequence database used for genomic surveillance
GP	Glycoprotein	Primary viral surface protein target for vaccines and mAbs
HCW	Healthcare Worker	Frontline medical personnel at risk of nosocomial infection
IHR	International Health Regulations	Legal framework defining global health security protocols
IND	Investigational New Drug	Regulatory status enabling emergency clinical evaluation
IPC	Infection Prevention and Control	Sanitary and administrative protocols to limit transmission
IQR	Interquartile Range	Statistical measure of distribution spread (25th to 75th percentile)
mAb / mAbs	Monoclonal antibody / antibodies	Target-specific immunoglobulins used for therapeutics
MeSH	Medical Subject Headings	Controlled vocabulary thesaurus used for indexing PubMed
MVA	Modified Vaccinia Ankara	Viral vector used in secondary prime-boost vaccine regimens
NHP	Non-Human Primate	Gold-standard animal model for filovirus research
PCC	Population-Concept-Context	Framework used to define scoping review eligibility criteria
PHEIC	Public Health Emergency of International Concern [12]	Formal declaration of global health emergency by the WHO
PMID	PubMed Identifier	Unique index number assigned to records in PubMed
PPE	Personal Protective Equipment	Specialized protective gear used by clinical personnel
PRISMA-ScR	Preferred Reporting Items for Systematic Reviews and Meta-Analyses extension for Scoping Reviews	Standardized reporting guidelines followed for this review
RCT	Randomized Controlled Trial	Gold-standard clinical trial design
RoB 2	Risk of Bias 2.0	Cochrane tool adapted to evaluate study quality
RT-PCR	Reverse Transcription Polymerase Chain Reaction	Gold-standard diagnostic laboratory assay
rVSV	Recombinant Vesicular Stomatitis Virus	Replicating vector platform used in the Ervebo vaccine
SBCC	Social and Behaviour Change Communication	Structured communication strategy to influence community practices
SIR	Susceptible-Infectious-Recovered	Compartmental mathematical modeling framework
SUDV	Sudan virus	Co-circulating filovirus species of comparative interest
TAFV	Taï Forest ebolavirus	Basal phylogenetically related filovirus species
WHO	World Health Organization	United Nations specialized agency for international public health
WGS	Whole-Genome Sequencing	Comprehensive genomic sequencing methodology

## Data Availability

The datasets analysed during the current study and analysis code are deposited on figshare at: https://figshare.com/s/e59037f09ddc7762865c.
